# Effect of Galangal Essential Oil Emulsion on Quality Attributes of Cloudy Pineapple Juice

**DOI:** 10.3389/fnut.2021.751405

**Published:** 2021-11-19

**Authors:** Wei Zhou, Yuefang Sun, Liqiang Zou, Lei Zhou, Wei Liu

**Affiliations:** ^1^State Key Laboratory of Food Science and Technology, Nanchang University, Nanchang, China; ^2^Key Laboratory of Tropical Crop Products Processing of Ministry of Agriculture and Rural Affairs, Agricultural Products Processing Research Institute, Chinese Academy of Tropical Agricultural Sciences, Guangdong, China

**Keywords:** galangal essential oil emulsion, cloudy pineapple juice, antimicrobial activity, physical stability, aroma profiles

## Abstract

Galangal essential oil is obtained from the rhizomes of galangal with proven anti-inflammatory, antioxidant, antiviral, and antimicrobial properties, which are valuable in the food industry. To explore the effect of galangal essential oil on the quality of pineapple juice, 0.05, 0.1, 0.2, and 0.4% galangal essential emulsion were added, and their influence on the physical stability, physicochemical properties, microbial quantity, and aroma profiles of cloudy pineapple juice were evaluated. The essential oil emulsion of galangal is a milky white liquid with a strong aroma of galangal. The pH values of emulsion increased from 4.35 to 5.05 with the increase in essential oil concentration, and there was no significant difference in the particle size of the pineapple juice. The results showed that the galangal essential oil emulsion was stable and the stability of the cloudy pineapple juice was significantly enhanced by the essential oil emulsion determined using LUMiSizer. The cloudy pineapple juice with a 0.2% essential oil emulsion showed the most stability during storage. The lightness of the cloudy pineapple juice increased instantly with the essential oil emulsion addition. In addition, the microbial quantity of the cloudy pineapple juice was decreased by the individual essential oil emulsion or combined with thermal treatment to hold a longer shelf life. The microbial counts in pineapple juice treated by 0.4% essential oil emulsion and thermal treatment only increased from 1.06 to 1.59 log CFU/ml after 4 days of storage at 25°C. Additionally, the pH and total soluble solids showed a slightly increasing trend; however, the value of titratable acidity, free radical scavenging capacity, and ascorbic acid content of the cloudy pineapple juice showed no significant change. Finally, the results of the electronic nose showed that the aroma components of the pineapple juice were changed by the essential oil emulsion and thermal treatment, and the difference was especially evident in the content of the sulfur, sulfur organic, and aromatics compounds. Consequently, the results indicated that galangal essential oil emulsion can be used as juice additives to improve the quality attributes and extend the shelf-life of cloudy pineapple juice.

## Introduction

Pineapple (*Ananas comosus*) is attributed to the *Bromeliaceae* family which is widely planted in many parts of the world (1–3). It is a commercially important tropical fruit and the high demand for cloudy pineapple juice is due to its distinct aroma, pleasant flavor, exquisite taste, and high nutritional values, especially abundant in ascorbic acid, polyphenols, and flavonoids (4, 5). Fresh cloudy pineapple juice is popular with consumers due to its bioactive compounds. However, the fresh cloudy pineapple juice has a short shelf life because of the enzyme activity, microbial spoilage, and physical factors during extraction, resulting in the loss of freshness and other nutrients. Therefore, to protect it from microbial contamination, product instability, and other damages, many physical and chemical methods have been adopted (6, 7). Due to the change of color, aroma, taste, and enzyme activity in processed fruit juices, the storage of fruit juices is restricted (8). In recent years, due to its safety and strong antibacterial activity, natural plant extracts and products have attracted more and more attention in controlling microbial pollution and maintaining fruit juice quality (9–13). Essential oils have been listed as generally recognized as safe compounds by the Unites States Food and Drug Administration (FDA), which have been approved as food flavors or additives (14). Previous research showed that cinnamon essential oil could be used to maintain the quality of apple juice (15). The study of the effects of the rosemary essential oil on the shelf-life of tomato juice showed that the rosemary essential oil capsules remained stable during the pasteurization of the tomato juice and maintained their antimicrobial activity (16). Furthermore, the assessment of the effects of the essential oil from betel leaf in apple juice revealed that compared with untreated juice, the shelf life of treated juice under refrigerated storage was extended by 6 days (17). Essential oil is effective for inhibiting and/or killing microorganisms in beverage systems, which is of great interest to the food industry.

Galangal, a perennial rhizomatous herbal plant widely grown in Southeast China, belongs to the Zingiberaceae family (3, 18). The rhizomes of galangal can be used as a traditional condiment to adjust food taste (2). The dried rhizomes of this plant can be used to cure stomachache, flatulence, and itching as an herbal medicine in China. According to recent research reports, galangal essential oil is chiefly composed of 1,8-cineole and other oxygen-containing monoterpenes (19, 20), which has been proved to have plenty of pharmacological effects such as antimicrobial, antioxidant, and anti-inflammatory (21, 22).

To our knowledge, researchers have studied the effects of essential oil emulsion on juice microbial quantity (23, 24). However, there is little research information about the effects of essential oil emulsion on the stability and flavor of fruit juices. Hence, attempts have been made to improve the shelf life of cloudy pineapple juice with essential oil from galangal. This study investigated the effects of galangal essential oil emulsion on the stability, physicochemical properties, microbial quantity, and aroma profiles of cloudy pineapple juice. This study aimed to assess the effect of galangal essential oil emulsion on cloudy pineapple juice quality, and it will be helpful to extend the shelf life of cloudy pineapple juice.

## Materials and Methods

### Materials

The pineapples were purchased from a local supermarket. The galangal essential oil was obtained from Guangdong Feng Xi Liang Jiang Co., Ltd (Zhanjiang, China). Ascorbic acid, 6-hydroxy-2,5,7,8-tetramethylchroman-2-carboxylic acid (Trolox) and 1,1-diphenyl-2-picryl-hydrazyl (DPPH) were purchased from Aladdin Chemicals Co. (Shanghai, China). Tween 80 and other chemicals were purchased from Sinopharm Chemical Reagent Co., Ltd. (Shanghai, China).

### Methods

#### Preparation of Galangal Essential oil Emulsion

The galangal essential oil emulsion was prepared according to the method of a previous study with a slight modification (15). The emulsion was mixed with 47 ml distilled water, 1 ml Tween 80, and 2 ml galangal essential oil. Using a high-shear mixer (ULTRA TURRAX® T18 digital, IKA, Staufen, Germany), we mixed the mixture which worked at 10,000 rpm for 5 min. Using distilled water, the 4% galangal essential oil emulsions were diluted to 0.5, 1, and 2% emulsions. The prepared emulsions were stored at 4°C before use.

#### Characterization of Galangal Essential oil Emulsion

The emulsions with different concentrations of galangal essential oil were characterized. The pH values of all emulsion samples were measured with a digital pH meter (FiveEasy Plus FE 28, Metller Toledo Instruments Co., Ltd., Shanghai, China). The appropriate amount of emulsion was diluted with deionized water of the same pH, then the particle size of the emulsion droplets was determined using NanoSizer (Malvern Instruments Ltd., Worcestershire, UK) (25). The polydispersity index (PDI) of the droplets was also analyzed with an average of 15 runs for each measurement at 25°C. All the measurements were performed at least three times on freshly prepared samples. The stability of the essential oil emulsion was evaluated using a LUMi-Sizer (L.U.M. GmbH, Berlin, Germany). The centrifugation speed was fixed at 500 rpm for 10 min at 25°C.

#### Preparation of Cloudy Pineapple Juice Samples

The pineapples were peeled, sliced, and juiced. Three layers of gauze were used to strain the cloudy pineapple juices. Then, different concentrations (0.5, 1, 2, and 4%) of galangal essential oil were added to the cloudy pineapple juice with the ratio of 1:9. The air in the glass bottles containing pineapple juice has not been emptied, and the volume ratio of the headspace to glass bottle was 1:7. Distilled water was added as a contrast. The final concentrations of emulsion in cloudy pineapple juices were 0.05, 0.1, 0.2, and 0.4%. All the treated samples were stocked at 4°C. Thermal treatments were conducted in a thermostat water bath. The juices were heated to 68°C for 30 min aiming at the destruction of pathogenic and spoilage microorganisms.

#### Dynamic Physical Stability of the Cloudy Pineapple Juice

The stability of the cloudy pineapple juice was measured using a LUMiSizer (L.U.M. GmbH, Berlin, Germany). The instability was promoted by centrifugal force. The sample was irradiated by near-infrared light to determine the function of the transmitted light intensity with time and position (26). The centrifugation speed was fixed at 500 rpm for 10 min at 25°C.

#### Color Measurements

The surface color (L^*^, a^*^, and b^*^ values) of the samples was evaluated using a colorimeter (CM-5, KonicaMinolta, JAPAN) set up for illuminant D65, 2° observer angle, and 3 s blooming time. The L^*^, a^*^, and b^*^ values represent the lightness, intensity of the red or green color, and intensity of yellow or blue color, respectively. Three independent replicates were performed for each juice sample (15).

#### Measurements of Physicochemical Properties

The total soluble solids (TSS), pH, and titratable acidity (TA) of the cloudy pineapple juice samples were determined. The TSS of the juice samples was independently evaluated with an Abbe refractometer (SW-LB20T, CHINA) at 25 ± 1°C which was expressed as °Brix. The refractometer prism was cleaned thoroughly with distilled water after performing each analysis. The pH value of the juice samples was evaluated using a digital pH meter. The pH meter was calibrated with commercial standard buffers pH 6.86 and pH 9.1 before measuring. The titratable acidity was measured according to the Association of Official Agricultural Chemists (AOAC) method which was expressed in the % citric acid (27). Titrate 20 g of cloudy pineapple juice with 0.1 mol/L NaOH to pH 8.2 for analysis. The % citric acid was calculated as the following equation:


$$\begin{array}{l} \%\text{citric acid}\left(\text{w}/\text{w}\right)=\frac{\text{volume NaOH }\times \;0.64\left(\text{ml}\right)}{\text{juice weight }\left(\text{g}\right)}\times 100\%\; \end{array}$$

Using the 2,6-dichlorophenol-indophenol (DCPIP) visual titration method determined the content of ascorbic acid in the samples (28). The cloudy pineapple juice was diluted with 20 g/L of metaphosphoric acid and then centrifuged. Then, the supernatant was titrated with standard dye solution (2,6-dichloroindophenol-indophenol and sodium bicarbonate) to a pink endpoint. The results obtained were expressed as the milligrams of ascorbic acid per 100 ml of the sample by the following equation:


$$\begin{array}{l} \text{Ascorbic acid content}\\ =\frac{\text{titer }\times \text{ dye factor }\times \text{ volume made up }\times \;100}{\text{aliquot of extract taken }\times \text{ volume of a sample taken}} \end{array}$$

The antioxidant capacity of the pineapple juice was evaluated by the 2.2-diphenyl-1-picrylhydrazyl (DPPH) method as described with slight adjustments. The pineapple juice (100 μL) diluted with 80% methanol was added to 2 mL of 0.1 mM DPPH and held in the dark for 1 h. The absorption of the samples (A_S_) was determined with an ultraviolet-visible spectrophotometer (UV-1600PC, MAPADA, Shanghai, China) at 517 nm and compared with a control sample without juice (A_C_). The antioxidant activity was expressed as percentage inhibition of the DPPH radical:


$$\begin{array}{l} \text{Percentage inhibition of DPPH}=\frac{A_{C}-A_{S}}{A_{C}}\times 100 \end{array}$$

#### Sensory Evaluation

The procedure performed for this sensory evaluation was based on the method reported by Mosqueda-Melgar et al. with minor modifications (29). The pineapple juice samples were evaluated by 30 experts (15 women and 15 men), including students and office staff between 20 and 45 years of age. The pineapple juice samples (50 ml) with different concentrations of galangal essential oil were served in cups coded with random numbers. Moreover, a glass containing water and a piece of non-salted biscuit was provided to the experts for removing the residual taste between the samples. Five different attributes were used to evaluate the overall quality using a 9 points scale (1, extremely dislike; 2, dislike very much; 3, dislike moderately; 4, dislike slightly; 5, neither like nor dislike; 6, like slightly; 7, like moderately; 8, like very much; 9, extremely like).

#### Microbiological Analysis

To evaluate the microbial quantity, the cloudy pineapple juice samples were evaluated with the total bacteria count and molds and yeasts method according to the procedure described by Zia et al. (30). The samples at 25 ± 1°C were diluted with sterile 0.9% sodium chloride (NaCl) solution. 1.0 mL of each dilution was plated into plates with a nutrient agar medium for the total bacteria count and potato glucose agar for the molds and yeasts. The plates were incubated at 37 ± 1°C for 48 ± 2 h for the total bacteria count and 28 ± 1°C for 5 days for molds and yeasts. After incubating, the plates with colonies between 10 and 130 could be used for counting. The LogN was calculated to determine the inhibition effect, where N (CFU/mL) was the number of viable microorganisms.

#### Electronic Nose Analysis

The aroma profiles of the cloudy pineapple juice samples were evaluated with an electronic nose system (PN3, Germany) as previously described, with minor modifications (31). Briefly, each sample (10 ml) was put into a 50 ml bottle and equilibrated for 5 min at 25°C under 500 rpm speed agitation. Before each analysis, the analysis system was purged with processed dry pure air. The headspace volatiles was put into the electronic nose for 10 s at a 400 ml/min rate. To obtain a stable signal, the acquisition duration for the sensors was 70 s. Each sample was tested six times to ensure the accuracy of the data, and the last three pieces of data were used for subsequent analysis.

#### Statistical Analysis

The experimental results were repeated three times. The values were presented as mean ± SD for each measurement. An ANOVA was performed for all the experimental runs to determine significance at 95% confidence with SPSS software (IBM, Armonk, New York, United States). A principal component analysis (PCA) was performed in Origin 2018 (Origin, Inc., San Francisco, California, United States) to describe the grouping of the aroma of cloudy pineapple juice based on the electronic nose data. The heat map was constructed and computed the hierarchical cluster analysis (HCA) based on Pearson's correlation. *P* < 0.05 was considered to be statistically significant.

## Results and Discussion

### Characterization of Galangal Essential oil Emulsion

The characterization of emulsion with the different concentrations of galangal essential oil is shown in Table 1. The pH values showed a decreasing trend with the increase in the emulsion concentration. The average particle size and polydispersity index of emulsion remained nearly unchanged as the galangal essential oil concentration increased from 0.5 to 4 %, indicating that the emulsions were stable under different concentrations of galangal essential oil (32). Meanwhile, the instability index of all the emulsions was low and significantly decreased as the concentrations of the galangal essential oil increased to 4%. Overall, the galangal essential oil emulsion was stable.

**Table 1:** The characterization of galangal essential oil emulsion.

**Emulsions (wt%)**	**pH**	**Average particle size (nm)**	**PDI**	**Instability index**
0.5	5.053 ± 0.018^a^	205.00 ± 3.52^a^	0.395 ± 0.014^a^	0.059 ± 0.001^a^
1	4.700 ± 0.006^b^	218.63 ± 9.93^a^	0.428 ± 0.009^a^	0.045 ± 0.000^b^
2	4.520 ± 0.007^c^	199.30 ± 2.44^a^	0.391 ± 0.005^a^	0.024 ± 0.001^c^
4	4.353 ± 0.078^d^	199.59 ± 3.27^a^	0.315 ± 0.019^b^	0.013 ± 0.001^d^

*Superscript letters indicate a significant difference between values in a given column (P < 0.05)*.

### The Physical Stability of Cloudy Pineapple Juice Samples

**Figure 1:**
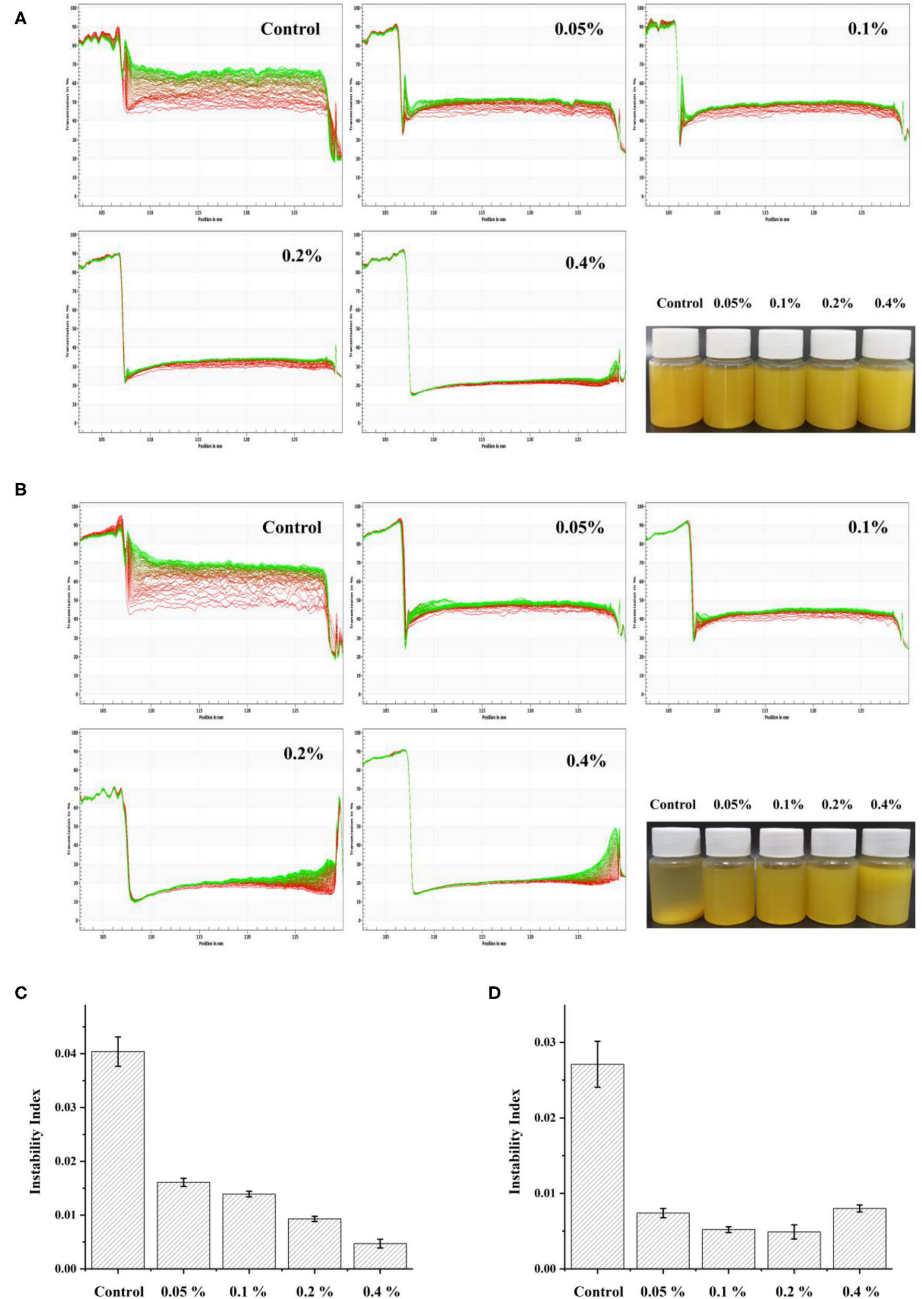
The transmission profiles of pineapple juice as measured by the LUMi-Sizer at 0 days **(A)** and 4 days **(B)**. The instability index of pineapple juice was measured by the LUMi-Sizer at 0 days **(C)** and 4 days **(D)**.

The cloudy pineapple juice sedimented easily, owed to the presence of suspended particles formed by polysaccharides, proteins, and phenols (33). A LUMiSizer analyzed the dynamic physical stability of the cloudy pineapple juice and the results are shown in Figure 1. After the centrifugation, the heavier phase moved down to the bottom of the test tube, which resulted in a more transparent aqueous phase (34). The red line was the first recorded contour, which then turned into the green line during the measurements. As shown in Figure 1A, the stability of the cloudy pineapple juice was significantly enhanced with the essential oil emulsion. The instability index of the cloudy pineapple juice sample was significantly decreased as the concentrations of emulsion increased to 0.4%. The juice with 0.4% essential oil emulsion was the most stable and the instability index was only 0.0047 after 0 days. After 4 days, the instability index of the cloudy pineapple juice sample decreased and then increased as the concentrations of the emulsion increased from 0.05 to 0.4%. At the concentration of 0.2%, the cloudy pineapple juice sample showed the best stability with the instability index of 0.0049.

The improvement of the physical stability of the cloudy pineapple juice by galangal essential oil emulsion is attributed to the thin interface layer composed mainly of emulsifier molecules. The relatively small particle size of the emulsion showed that Brownian motion effects played a central role. Thus the emulsion had high gravitational separation stability (15, 35). In addition, with the increase of storage time, the essential oil emulsions may aggregate and float due to excessive concentration, which resulted in the decreased stability of the juice. Therefore, the galangal essential oil emulsion in the appropriate concentration range might prevent the sedimentation of particles in pineapple juice to improve its physical stability.

### The Color Changes of Cloudy Pineapple Juice Samples During Storage

**Figure 2:**
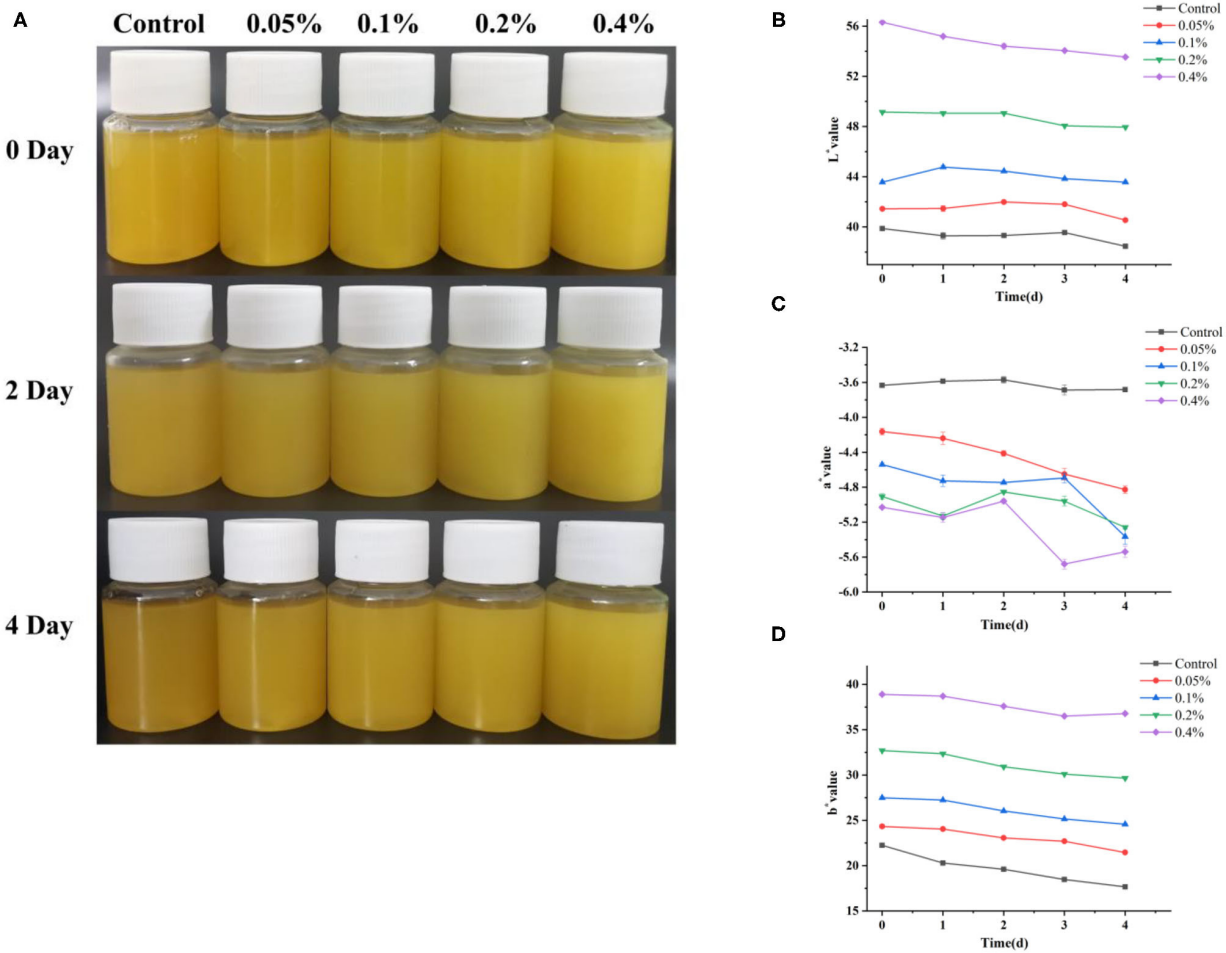
The results regarding the visual appearance of the pineapple juice during storage at 4°C **(A)**. The changes in L^*^ **(B)**, a^*^ **(C)**, and b^*^ **(D)** values of pineapple juice during storage.

Color may be determined or influenced by certain factors during storage. Figure 2 shows the effects of emulsion concentration and storage time on juice color. The appearance of the cloudy pineapple juices during storage at 4°C is shown in Figure 2A. During storage after 0 days, the L^*^, b^*^ values of the cloudy pineapple juice with the different concentrations of essential oil emulsion were increased significantly. On the contrary, the a^*^ value of the cloudy pineapple juice was decreased. As the essential oil emulsion itself is milky white, the lightness of the processed fruit juice will increase instantly. However, the slight browning of the cloudy pineapple juice treated with essential oil emulsion was observed after 4 days. In general, the color change of the cloudy pineapple juice during storage is mainly due to enzymatic browning in which the key enzyme is polyphenol oxidase (PPO) in many fruits (36, 37). Polyphenol oxidase results in the browning of damaged vegetables and fruits by converting *o*-diphenols and *o*-diphenols to *o*-quinones in plants, which polymerize from brown pigments subsequently (38). Stewart et al. found that PPO activity was low in the pineapple roots, leaves, and developing and mature fruit (39). Additionally, the PPO activity in the harvested pineapple fruits was very low and remained low during 4 weeks of storage at 25°C (40), which may be the main reason for the color change of the pineapple juice during storage.

### The Change of Physicochemical Properties and Sensory Characteristics of the Cloudy Pineapple Juice

The physicochemical properties of pH, TSS, TA, DPPH free radical scavenging capacity, and ascorbic acid content in the cloudy pineapple juice after adding the essential oil emulsion are shown in Table 2. The pH values increased slightly after adding the essential oil emulsion, but all the values were kept at 3.2–3.4. Previous studies reported that the pH of raw apple juice samples was not affected by betel leaf essential oil emulsion (17). Due to the different pH values of the essential oil emulsion from 4.35 to 5.05 in this study, the pH values of the cloudy pineapple juice slightly changed. The TSS values of the cloudy pineapple juice also showed a slightly increasing trend, probably due to the stability of the essential oil emulsion for gravitational separation (41). However, for the value of TA, DPPH, and ascorbic acid content, there were no significant differences between the cloudy pineapple juice samples. Similar results were observed in a previous study wherein the addition of *Mentha arvensis* L or *M. piperita* L to cashew, guava, mango, or pineapple juices showed no alteration in TA (23). In addition, Suradeep Basak also found that the antioxidant capacity of apple juice samples by betel leaf essential oil was not changed (17).

**Table 2:** Changes in the physicochemical properties (pH, TSS, TA, DPPH, Vc) of the pineapple juice treated by galangal essential oil emulsion at room temperature.

**Emulsions (wt%)**	**pH**	**TSS (^**°**^Brix)**	**TA (% CA)**	**DPPH (% μg/ml TE)**	**Vc (mg/100g)**
Control	3.286 ± 0.003^c^	11.23 ± 0.03^d^	0.595 ± 0.012^a^	25.05 ± 0.18^a^	13.67 ± 0.16^a^
0.05	3.276 ± 0.003^c^	11.33 ± 0.03^cd^	0.619 ± 0.014^a^	24.78 ± 0.14^a^	13.74 ± 0.07^a^
0.1	3.343 ± 0.003^b^	11.46 ± 0.03^bc^	0.595 ± 0.008^a^	24.62 ± 0.02^a^	13.51 ± 0.07^a^
0.2	3.356 ± 0.003^ab^	11.50 ± 0.00^b^	0.604 ± 0.001^a^	24.73 ± 0.07^a^	13.20 ± 0.07^a^
0.4	3.370 ± 0.005^a^	11.70 ± 0.03^a^	0.600 ± 0.022^a^	24.84 ± 0.15^a^	13.28 ± 0.15^a^

*Values are mean ± SE (n = 3), and columns with different letters are significantly different (p < 0.05)*.

*TSS, total soluble solids; TA, titratable acid; CA, citric acid; DPPH, 1,1-diphenyl-2-picrylhydrazyl radical; TE, Trolox equivalent; Vc, ascorbic acid content*.

The influence of the cloudy pineapple juice with or without galangal essential oil emulsion on the sensory evaluation concerning aroma, color, taste, sourness, and overall acceptability is shown in Table 3. Compared with the untreated samples, no significant changes in the taste and sourness of all the cloudy pineapple juices were observed. With the addition of the concentration of essential oil emulsion, the scores of color increased significantly. However, the essential oil emulsion at a concentration higher than 0.05% caused negative effects on the aroma and overall appearance. Overall, the cloudy pineapple juice with a 0.05% essential oil emulsion was the most acceptable concerning the aroma and overall appearance. According to previous studies, Behnoush Maherani et al. observed a similar result that 0.01% of plant oil microemulsion had no negative effect on the aroma and taste of orange juice (42). Additionally, 0.02% orange essential oil microemulsion resulted in a slightly lower overall acceptability value in apple juice while the difference was not significant (43). Furthermore, Marta Laranjo et al. found that plant essential oils, such as cinnamon, sage, clove, rosemary, oregano, and thyme did not depreciate the sensory quality in cheeses, however, oregano essential oil presented a pronounced bitter taste (11). To summarize, the addition of essential oil emulsion might mainly negatively affect the aroma and a low concentration was recommended for fruit juice. Moreover, the balance between sensory quality and food safety after the addition of essential oil needs to be further investigated.

**Table 3:** Effect on the organoleptic characteristics of the pineapple juices treated under galangal essential oil emulsion.

**Emulsions (wt%)**	**Aroma**	**Color**	**Taste**	**Sourness**	**Overall**
Control	7.5 ± 0.2^b^	6.5, 0.3^c^	7.6 ± 0.3^a^	6.5 ± 0.6^a^	7.7, 0.4^ab^
0.05	8.0 ± 0.5^a^	6.8, 0.4^bc^	7.4 ± 0.2^a^	6.4 ± 0.4^a^	8.0, 0.3^a^
0.1	7.1 ± 0.5^b^	7.0, 0.5^b^	7.4 ± 0.3^a^	6.4 ± 0.3^a^	7.4, 0.3^bc^
0.2	6.6 ± 0.4^c^	7.5, 0.4^a^	7.4 ± 0.7^a^	6.5 ± 0.6^a^	7.3, 0.4^c^
0.4	6.0 ± 0.5^d^	7.6, 0.3^a^	7.4 ± 0.2^a^	6.4 ± 0.4^a^	7.1, 0.4^c^

*Different lower-case letters (a, b, c, d) on the same column indicate SDs (P ≤ 0.05) among processes by each sensory attribute and fruit juice*.

a *Values are the mean of 30 evaluations ± SD*.

### The Change of Antibacterial Activity During Storage and Thermal Treatment

**Figure 3:**
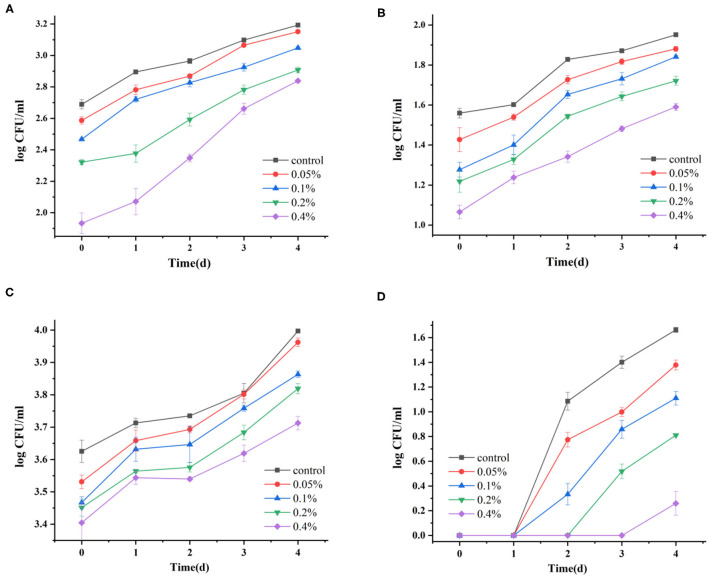
The change of the total bacteria count, molds, and yeasts in pineapple juice. **(A)** The change of the total bacteria count by essential oil emulsion. **(C)** The change of molds and yeasts by essential oil emulsion. **(B)** The change of the total bacteria counts by essential oil emulsion and heat treatment. **(D)** The change of molds and yeasts by essential oil emulsion and heat treatment. **(A,C)** stored at 4°C. **(B,D)** stored at 25°C.

Essential oils such as galangal had potent antibacterial efficacies and are generally considered safe for food application. As shown in Figure 3, the growth of the total bacteria count, molds, and yeasts was inhibited after the essential oil emulsion and thermal treatment. Taken as a whole, the number of microbial counts increased with progress in the storage period. With the increase in the essential oil emulsion concentration, the total bacteria count, molds, and yeasts decreased significantly (Figures 3A,C). On day 0, the count of the total bacteria, molds, and yeasts were 1.77 and 3.40 log CFU/ml with 0.4% of essential oil emulsion, respectively. According to previous studies, essential oils as antibacterial agents can effectively reduce microorganism spoilage such as fungi and yeasts (16, 44). The decrease of the microbial populations might be attributed to the protection of the essential oil, gradually released from capsules. *Suradeep Basak* investigated the preservative effect of betel leaf essential oil microemulsion on improving the safety and sensory attributes of apple juice, which showed that raw apple juice treated by betel leaf essential oil microemulsion was extended by 6 days as compared with untreated juice under refrigerated storage (17). Compared with the yeast and mold growth, the investigated essential oil showed a better inhibitory effect on bacterial pollution.

After the thermal treatment, compared with the juice with essential oil emulsion, the total bacteria count, molds, and yeasts in the cloudy pineapple juice with essential oil emulsion were inhibited significantly. Interestingly, no molds and yeasts were observed after the thermal treatment until 3 days of storage, while this treatment did not effectively prevent the growth of total bacteria count in the initial state. The inhibition effect of the thermal treatment on molds and yeasts was better than the total bacteria count. In addition, Roberta Bento et al. also reported that combined mild heat with essential oil emulsion was an advantageous alternative to preserve fruit juice (43). The cloudy pineapple juice quality and shelf-life extension can be improved by combining essential oil emulsion with thermal treatment.

### The Overall Assessment of Cloudy Pineapple Juice Based on HCA

**Figure 4:**
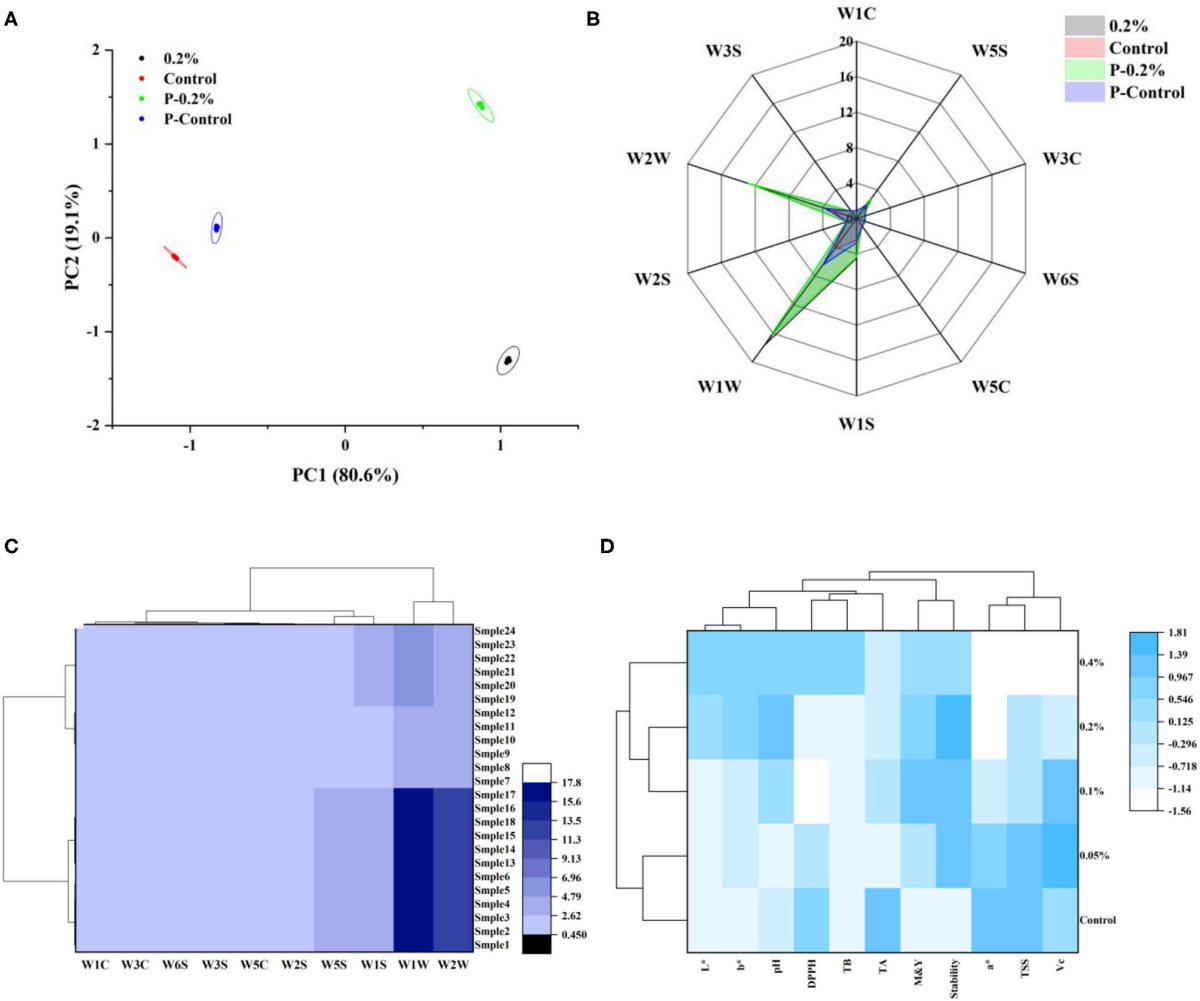
Flavor analysis of four pineapple juices: Principal component analysis (PCA) of volatile flavor by the electronic nose **(A)**; radar image of smelling determination by the electronic nose **(B)**; hierarchical cluster analysis (HCA) based on flavor **(C)**. “P-” was the heat treated sample. Heatmap combined with dendrogram of cluster analysis **(D)**. “TB” was total bacteria count; “M&Y” was molds and yeasts.

An HCA method was applied to treat five pineapple juice samples based on their measured properties. Each variable was standardized before the treatment. Moreover, a heat map showed how the five juices varied in properties. The processing and production technology of apple juice was analyzed using the same method (45). The heatmap is shown in Figure 4D. Regarding the HCA, all the juice samples were grouped into two clusters. The clusters demonstrated that the pineapple juice with 0.1, 0.2, and 0.4% essential oil emulsion were closely grouped, possessing higher values of L^*^, b^*^, pH, DPPH, total bacteria count, molds and yeasts, and physical stability. Additionally, the pineapple juice with the control and 0.05% essential oil emulsion were clustered into the same group for their relatively higher value of a^*^, TSS, and Vc. In terms of their antimicrobial activity and physical stability, a better quality of pineapple juice was observed with a higher concentration of essential oil content.

### The Difference of Cloudy Pineapple Juice Aroma Profiles

A series of complex volatile substances causes the sensations of smell. The electronic nose is an analytical instrument imitating the human system and a nondestructive testing method whose detection process is fast and straightforward (31). An electronic nose analysis was carried out for the samples to differentiate the aroma profiles of the cloudy pineapple juice samples. The PCA determined the pattern recognition between the essential oil emulsion and the complex aroma of the cloudy pineapple juice as shown in Figure 4A. The better difference of the groups was observed with the more excellent dispersion between data collection points (3). There was no obvious overlap of volatile aroma data collection points among the different cloudy pineapple juice varieties, indicating that PCA could better distinguish the aroma. The principal components (PC1 and PC2) accounted for 80.6% and 19.1% of the total variance, respectively. These two PCAs together exceeded 99% of the information in the total data set. The PCA conducted displayed that the position of “0.2%” and “P−0.2%” was far away from the origin of the vertical coordinate axis, and separated from each other, which showed that they were different from each other on volatile aromas.

Figure 4B is a radar diagram that shows the 10 sensor response values of the electronic nose for each of the four samples; each line means the changes in the relative resistivity of one sensor (46). The electronic nose showed that W1W, W2W, W5S, and W1S were relatively sensitive to the sample gas by combining essential oil emulsion with thermal treatment. The sensors W1W, W2W, W5S, and W1S are sensitive to sulfur compounds, sulfur organic compounds, and aromatics compounds, nitrogen oxides, and methane, respectively (47). The HCA of four kinds of cloudy pineapple juice was conducted (Figure 4C) and revealed the relationships between the cloudy pineapple juice and the sample aroma indicators. The HCA grouped samples in clusters, using all samples simultaneously based on their similarities calculated from the distances between samples (48). The sensory evaluations were clustered from the four samples and their similarities were summarized. The samples were divided into two categories and four subcategories, consistent with the results in Figure 4A. In summary, essential oil emulsion and heat treatment changed the aroma components of the cloudy pineapple juice, which increased the content of sulfur compounds, sulfur organic compounds, and aromatics compounds.

## Conclusions

This study investigated the effect of galangal essential oil emulsion on the quality attributes of cloudy pineapple juice. It was found that galangal essential oil emulsion was stable. The essential oil emulsion improved the physical stability of cloudy pineapple juice and the stability of the cloudy pineapple juice with a 0.2% essential oil emulsion was the best after 4 days of storage. The lightness of the cloudy pineapple juice increased immediately as soon as the essential oil emulsion was added, but slight browning was observed after 4 days of storage. In addition, the physicochemical properties, antioxidant capacity, and Vc content of the cloudy pineapple juice showed no significant changes. The microbial quantity of the cloudy pineapple juice was reduced by combining essential oil emulsion with thermal treatment. Finally, the aroma profiles of the cloudy pineapple juice were changed by essential oil emulsion and heat treatment, increasing the content of sulfur compounds, sulfur organic compounds, and aromatics compounds. This study revealed the effect of galangal essential oil emulsion with quality indicators of cloudy pineapple juice and suggested the potency of galangal essential oil as a juice preservative. Therefore, galangal essential oil emulsion treatment can be considered as a promising non-thermal alternative for microbial decontamination and the improved quality of fresh cloudy juice. However, further research on the effect of galangal essential oil on different juice qualities should be conducted to ensure the efficacy of galangal essential oil as a food additive.

## Data Availability Statement

The original contributions presented in the study are included in the article/supplementary material, further inquiries can be directed to the corresponding author/s.

## Author Contributions

WZ: conceptualization, investigation, data curation, and writing original draft. YS: investigation, data curation, and writing original draft. LZo: validation, methodology, writing review, and editing. LZh and WL: supervision and project administration. All authors have read and agreed to the published version of the manuscript.

## Funding

This research was financially supported by the Research and development project in key areas of Guangdong Province (2020B020225003); National Science Foundation of China (No. 31860452); Research Project of State Key Laboratory of Food Science and Technology, Nanchang University (No. SKLF-ZZB-201919); Supported by the Open Project Program of State Key Laboratory of Food Science and Technology, Nanchang University (No. SKLF-KF-202015), and Foundation of Key Laboratory of Tropical Crop Products Processing of Ministry of Agriculture and Rural Affairs, China (KFKT202002).

## Conflict of Interest

The authors declare that the research was conducted in the absence of any commercial or financial relationships that could be construed as a potential conflict of interest.

## Publisher's Note

All claims expressed in this article are solely those of the authors and do not necessarily represent those of their affiliated organizations, or those of the publisher, the editors and the reviewers. Any product that may be evaluated in this article, or claim that may be made by its manufacturer, is not guaranteed or endorsed by the publisher.
